# Addressing issues of vaccination literacy and psychological empowerment in the measles-mumps-rubella (MMR) vaccination decision-making: a qualitative study

**DOI:** 10.1186/s12889-015-2200-9

**Published:** 2015-09-02

**Authors:** Marta Fadda, Miriam K. Depping, Peter J. Schulz

**Affiliations:** Institute of Communication and Health, Faculty of Communication Sciences, University of Lugano, Via G. Buffi 13, 6900 Lugano, Switzerland

## Abstract

**Background:**

Whether or not to vaccinate one’s child is one of the first health-related decisions parents have to make after their child’s birth. For the past 20 years, the share of parents choosing not to immunize their children has increased in many countries, for various reasons. Among these, rumors affirming that vaccinations contain dangerous chemicals or might trigger severe chronic diseases have negatively affected parental attitudes towards pediatric immunizations, particularly the vaccination against measles, mumps and rubella (MMR), raising a number of public health concerns. The primary aim of this qualitative study is to understand what drives parents’ decision, giving special attention to vaccination literacy and psychological empowerment in such a context.

**Methods:**

Twenty individual semi-structured interviews were conducted in the Canton of Ticino (Switzerland) between January and June 2014. Participants were either mothers or fathers of children less than 1 year old living in Switzerland. An inductive thematic analysis was performed to identify the main themes with regard to vaccination literacy and psychological empowerment in the MMR vaccination decision-making.

**Results:**

Parents’ reports yielded four main themes: (a) the paradox of the free choice, referring to the misinterpretation of current vaccination policies; (b) giving up the power, pointing at the outcomes of a low perceived competence; (c) a far-reaching decision, reflecting the importance attributed to the MMR choice and the different levels of impact the decision can have; (d) the demand for shared-decision making, referring to the parental needs in relation to the child’s healthcare provider.

**Conclusion:**

Understanding what drives parents’ management of their children’s immunization schedule in terms of vaccination literacy and psychological empowerment can help health professionals to communicate more effectively with parents in order to facilitate an informed decision, and stakeholders to design tailored health education programs and materials. This can ultimately help increase the coverage of the MMR vaccination.

**Electronic supplementary material:**

The online version of this article (doi:10.1186/s12889-015-2200-9) contains supplementary material, which is available to authorized users.

## Background

Measles is an infectious respiratory disease, which can lead to severe complications particularly in children under the age of 5 and adults over the age of 20 [1]. In developing countries, measles is still one of the leading causes of death among children, although a safe, efficient and relatively inexpensive two-dose vaccination is available [2]. The most common measles-containing vaccine is the MMR vaccine, which also protects from mumps, a disease characterized by swelling of the salivary glands, and rubella, an infection that can often lead to serious complications in the fetus if acquired by an expecting mother [1]. To reach herd immunity, health authorities recommend that 95 % of the population be vaccinated [2].

In most developed countries, parents are recommended to immunize their children against MMR, but the final decision is theirs. This policy, which calls for an informed, autonomous decision, assumes parents possess the relevant and accurate information regarding both the risks and the benefits of the vaccination compared to the disease, the skills to judge what is more appropriate for their child, and the motivation to engage autonomously in such a decision. In other words, parents are expected to be knowledgeable and empowered in order to make their choice, whether or not their final decision will meet the country’s official recommendations. Indeed, even with a sound knowledge and a high level of engagement in the decision-making, different factors and cognitive processes might lead to a biased judgment, such as omission biases [3]. Although making vaccination compulsory may be seen as a strategy to boost adherence to vaccination programs, compliance with vaccination schedules in Europe is high even when vaccinations are merely recommended [4, 5].

As in most European countries, the MMR vaccination is not compulsory in Switzerland. The country is committed to the goal of eliminating measles and rubella in the European Region of the World Health Organization by 2015. However, it currently displays suboptimal MMR coverage, making measles still locally endemic [6–8]. Recent data from the Swiss Federal Office of Public Health (FOPH) show that only 86 % of 2-year-old children have received the two doses that make a full MMR course [9].

Between 2006 and 2009, Switzerland experienced the highest measles incidence rate of Central and Western Europe, making up 29 % of all measles cases that occurred in the 32 European countries reporting to the same surveillance network (ECDC) [6]. Despite a widespread prevention campaign, measles cases in Switzerland have nearly doubled in 2013 compared to the previous year [9]. In addition, Switzerland constitutes a potential source of imported measles for other countries in Europe and elsewhere, such as Germany, Denmark, England, and the United States [6].

Research has extensively studied drivers and barriers of parental vaccination decisions. The most significant predictors of vaccination behavior include perception of the risks posed by the disease and the vaccination side effects [10–14], beliefs and attitudes towards the disease and the vaccination [15–19] and its efficacy [20], and safety concerns [21–23]. An extensive literature has also acknowledged the role of trust in medical professionals, health authorities, and governments [13, 24–29], and social norms [30]. In addition, religious beliefs [31], hesitancy [32], publicity by anti-vaccination groups [33–36] and the rise of complementary and alternative medicine (CAM) have been reported as playing a crucial role [37–40]. The pediatrician’s information [41] and communicative style during vaccination recommendation (presumptive vs. participatory) [42] can also be influential on the decision. Mixed results are available for the role of demographic variables such as education [43–46], age, race, marital status and number of children [47–49]. Furthermore, evidence suggests that immigrants are more likely to adhere to vaccination recommendations compared to the local population [50–52]. Knowledge has also been identified as an indirect driver [10, 43, 53–56].

Within the extensive literature currently available on what informs parental decision regarding childhood vaccinations, several studies have specifically looked at the context of the MMR vaccination, especially after the MMR scare sparked by a Lancet article which claimed a link between MMR and autism in 1998 [49, 57–59]. A summary of the most common factors underlying parental MMR vaccination decision making can be found in a recent systematic review [60].

Research has shown that a unique set of beliefs and different positive and negative attitudes surround each vaccination and its related disease(s) [29]. Our study aims to explore the reasons that drive parents’ MMR vaccination decision, with a careful look at vaccination literacy and psychological empowerment. To our knowledge, this is the first study addressing vaccination literacy and empowerment together in the context of parents’ decision to have their child immunized or not. The MMR vaccination features a number of unique characteristics compared to other childhood vaccinations – such as being at the center of the autism controversy [61]. Moreover, administering this vaccine can be seen by parents as the closest thing to a natural infection, since it is made of live attenuated viruses of its three target diseases [62]. Studies have also shown that postponing this vaccination may have serious consequences for future outbreaks [63].

### Theoretical background

Since parents have the final say on their children’s immunization, the MMR vaccination decision is extremely sensitive to individual differences. A number of theories have addressed such behavioral differences from a variety of perspectives. Among these, the Health Empowerment Model provides a theoretical framework that considers health literacy and psychological empowerment as two equally important and independent predictors of health behavior [64]. The model has been applied to a number of contexts, including eHealth interventions [65] and studies on chronic patients’ self-management [66]. Recently, its application to the context of vaccination behavior has been advocated to explain parental resistance against physicians’ professional standards, suggesting the potential danger of vaccination misinformation when this is coupled with high parental empowerment [64].

Nutbeam [67] defines health literacy as “the capacity to acquire, understand and use information in ways which promote and maintain good health”. Schulz & Nakamoto [64] stress the multidimensionality of this concept, defining it as a set of four sub-dimensions: (a) functional literacy, (b) declarative knowledge, (c) procedural knowledge, and (d) judgment skills. Similar to health literacy, psychological empowerment is an intrinsic motivational construct of the individual manifested in four cognitions [68–70]: (a) meaningfulness (the extent to which what one does is perceived as being important), (b) competence (one’s perceived competence to carry out an action), (c) impact (the perception of making a difference through a certain action) and (d) self-determination (the extent to which what we do is perceived as autonomous). Although the term empowerment originally focused on the individual, the collective, and the organizational levels [71], our study shall be concerned with the individual level only. Ideally, people will possess the adequate knowledge and skills to manage their own care, but also the commitment and motivation to make autonomous and impactful decisions. For a more thorough description of the Health Empowerment Model, see Schulz and Nakamoto [64].

In the context of parental vaccination decision, health literacy can be studied in terms of both knowledge about vaccinations and ability to find, judge and use the information encountered, in light of the high amount of inaccurate material which parents can be exposed to [72]. Knowledge can be further split into declarative and procedural. Declarative knowledge includes, for instance, knowledge about infectious diseases, the availability of vaccines, or the likelihood and severity of their side-effects. Procedural knowledge entails notions such as knowing how and when to get vaccinated against infectious diseases [73].

Adjusted to the context of parental vaccination decision-making, the four sub-dimensions of psychological empowerment can be operationalized as following: (a) meaningfulness will refer to the degree to which an individual thinks that making a vaccination decision regarding his or her child is an important issue; (b) competence will refer to the degree to which an individual feels able to make a sound vaccination decision; (c) impact will refer to the degree to which an individual feels that making a decision over the vaccination can generate a number of outcomes; (d) self-determination will refer to the degree to which individuals think that their vaccination decision is solely determined by themselves. A study was conducted using semi-structured interviews with parents in order to explore the factors driving parental MMR vaccination decision with regards to vaccination literacy and psychological empowerment.

## Methods

### Recruitment and participants

Qualitative methods are most appropriate when a better understanding of a phenomenon is sought [74], or when a theory needs to be built. Individual interviews rather than focus groups were chosen as they allow to obtain a deeper individual understanding of parents’ vaccination literacy and empowerment in the MMR vaccination decision making.

Participants were recruited in the Canton of Ticino (Italian-speaking Switzerland). To maximize the variability of our sample’s experiences, we employed a diverse recruiting system. Invitation flyers were sent to pediatricians and gynecologists, distributed at local public and private nursery schools, pre-schools, supermarkets, pharmacies, yoga and baby splash classes. In addition, invitations were circulated in a number of public spaces and printed in a number of local newspapers. Participation was optional and participants received a 20.- CHF shopping voucher as compensation.

Eligibility criteria for this study included: (a) being parent of at least one child under the age of 12 months (since the administration of the first dose of the MMR vaccination is recommended in Switzerland when the child turns 1-year-old, our inclusion criteria allowed to meet parents during their vaccination decision-making); (b) being a permanent resident in the Canton of Ticino.

### Ethics

The Ethical Committee of Canton Ticino gave ethical approval to the study (Rif. CE 2770). Each participant signed a consent form prior to the interview.

### Data collection

We conducted 20 face-to-face, semi-structured individual interviews in Italian, which lasted approximately 30 min each. We used semi-structured interviews in order to have a flexible grid of structural and open questions, allow each interviewee to describe his or her experience and introduce new themes spontaneously. The interview guide was developed on the basis of the Health Empowerment Model [64] to elicit detailed information on: (1) confidence in one’s MMR vaccination decision; (2) vaccination literacy, including general beliefs, procedural knowledge, subjective knowledge, perceived outcomes of MMR, and information-seeking behaviors [75, 76]; (3) psychological empowerment, according to its conceptualization into the four sub-dimensions of meaningfulness, impact, self-efficacy, and self-determination [64]; (4) social influences; (5) reactions to MMR-related information; (6) usage of complementary and alternative medicine (CAM); (7) risk perception of both measles and MMR side effects (comprising severity and susceptibility of their respective consequences); (8) barriers to the decision. See Additional file 1 for a detailed interview schedule containing all questions.

The vast majority of the interviews were conducted by the first author, who has a background in social anthropology, either at parent’s house, workplace, or at the University, according to their preference. To assess children’s age and collect parents’ socio-demographic characteristics (age, origin, education, number and age of children), a short questionnaire was sent by email to each participant upon completion of the interview.

If participants explicitly gave us permission, they were sent official information leaflets on measles and the MMR vaccination together with the gift card and a debriefing letter after the interview.

Data collection and data analysis were carried out simultaneously over a period of 5 months beginning in January 2014. Data collection ceased once data saturation was reached, that is when it was decided that additional interviews would not yield new data, but only confirm what was found in previous interviews [77].

### Analysis

Each interview was recorded, using a digital voice recorder, and transcribed verbatim by the main researcher and the research assistant (both native Italian speakers) within 3 days from completion of the interview. The transcripts were read several times by the main researcher to become familiar with the content, and they were later entered into NVivo for the coding (QSR International Pty Ltd. Version 10, 2012). Both transcription and analysis of the interviews were conducted in the original language (Italian) to avoid missing significant elements during the translation process. An inductive thematic approach [78] was used for the analysis of the data. Meaningful utterances were grouped and later categorized under several labels. Labels were subsequently organized hierarchically [79], and similar labels were then gathered into bigger themes. Preliminary themes, labels and utterances were then discussed with two senior qualitative researchers who provided feedbacks in relation to the ongoing analysis. At the end of this process, all transcripts were read again to establish logical links between different themes. The results of the inductive thematic analysis will be described in the following section, while they will be interpreted in the discussion section by making the link to our research question.

## Results

### Participant characteristics

Demographic data are summarized in Table 1. Most participants were mothers, had more than one child, and were in their thirties (age range 23–42). Regarding education, either a university degree or a professional university certificate was held by nearly two thirds of the sample. Immigrants represented a large percentage of our sample, which is in line with current statistics about the migrant population in Switzerland (estimated at 35 % [80]). On the basis of their reports, participants were classified as either being opposed (*n* = 3), favorable (*n* = 13), or undecided (*n* = 4) with regards to the MMR vaccination.

**Table 1:** Characteristics of the participants

*Total number*	20 participants
*Gender*	15 mothers and 5 fathers
*Age*	23–42 years old (*M* = 34)
	3 participants: ≤29 years old
	14 participants: 30–39 years old
	3 participants: ≥40 years old
*Origin*	13 from EU, 2 from non-EU countries, 5 from Switzerland
*Level of education*	2 secondary school, 4 high school education or equivalent, 14 university or professional university degree
*Number of children*	6 participants: 1 child
	12 participants: 2 children
	2 participants: 3 children
*Attitude towards the MMR vaccination*	3 participants: opposed
	13 participants: favorable
	4 participants: undecided

The analysis of the transcripts yielded four main themes: (a) the paradox of free choice, (b) giving up power, (c) a far-reaching decision, and (d) the demand for shared-decision making. Parents’ perceptions with regards to the likelihood to catch measles varied across the participants. Most parents agreed that measles is a highly infectious disease that can spread even faster if the child frequents other children, and learned from different sources that the disease is “making a comeback”. Undecided and vaccination-opposed parents, on the other hand, believed that their children were not likely to catch it, and expressed a preference for either natural immunity or safer alternatives to the MMR vaccination as a form of prevention. Only few, highly educated pro-vaccination participants cited the possible serious consequences of measles. The majority of parents found, instead, that measles was not a serious disease, referring to it as a type of “chickenpox” that can only have serious consequences in adults. Experience seemed to shape the perception of the severity of measles among those participants who had contracted the disease in the past. Pro-vaccination parents felt that their children were not likely to incur in side-effects due to the vaccination and they did not consider them to be serious, while undecided and opposed participants perceived them as highly probable and severe.

### The paradox of the free choice

Unlike some of its neighboring countries [4], Switzerland does not have any mandatory vaccinations, but parents are recommended to follow a specific vaccination schedule for their children. The MMR vaccination is among the recommended vaccinations. However, a main finding was that parents differed in their interpretation of current vaccination policies in Switzerland with regards to the MMR vaccination, with some misinterpreting the ultimate scope of the free choice in the vaccination decision, i.e. parental empowerment. The view that MMR would be mandatory if measles were a serious disease, and not merely recommended by health authorities, dominated the reports by vaccination-opposed parents.

*“I say, if it was really a serious disease that has to be absolutely eradicated and never appear again, I believe all nations would agree on vaccinating children. […] When they asked me if I wanted to vaccinate him against rubella, I did not feel like it. I said no. […] I listened to their opinion, but I did not listen to their advice. In the end, I decided to follow what my husband and I had decided to do”.(Mother, 38, Ticino, Secondary School, Opposed)*

The same mother, guided by her perception that catching measles was not likely, expressed a preference for either natural immunity or safer alternatives to the MMR vaccination as a way to prevent her child from getting the disease:

*“If there was a measles outbreak somewhere… well, I would pay more attention. But I have the impression that everything always works by hand contact, doesn’t it? I have this idea in mind, that if I teach him (the child) to regularly wash his hands, since he also likes water a lot, he will be protected… This is my prevention”.*

Some parents believed that Switzerland had both compulsory and recommended vaccinations, and translated the non-compulsoriness of the MMR vaccination as further evidence that it was not a necessary preventive measure:

*“Anyway, I said, let’s do the basic ones, the almost mandatory ones, those. Whether I want or not? I don’t want! Because if you tell me ‘if you want’ it seems optional, an optional vaccination, for me it seems there is no risk, no? If it is optional… come on! [..] And since I will make the decision with my wife, we often go and look for information. We look on the Internet, we only look on the Internet”.(Father, 28, non-EU, University, Opposed)*

On the other hand, parents that had a positive attitude towards the MMR vaccination saw current policies as a sign that the vaccination is important to protect children from unnecessary illness.

*“If they offer a vaccination, there must be a reason. I do not want him to get a disease that is out there. Vaccinating is life”.*

*(Father, 35, Non-EU, Obligatory School, Favorable)*

### Giving up the power

A number of parents reported that they perceived themselves to be unable to make a sound decision for their child. As a consequence of this feeling, some of them reported that they gave up their role as the agent in the management of their child’s health, while others opted for an autonomous decision anyway. Some completely relied on other decision-makers such as the pediatrician or followed what their parents had done with them or was prescribed in their original culture, while others made a gut-driven decision. To some parents, ability to make a decision included the skills needed to grasp the official information received by health authorities and health professionals. As this language mainly includes statistical information on the likelihood of getting the disease or experiencing vaccination adverse events, parents reported a preference for narrative information on the MMR vaccination, which they described as easier to understand.

*“Moreover, my problem is that I don’t have a scientific background. And when you hear… When you read this* [official] *information, you realize they all start from the results of some statistical tests that they did on vaccinations. Maybe the base is wrong… because one starts from certain statistical data, and the other starts from the same, but not keeping into account other data”.(Mother, 34, EU, Professional University, Opposed)*

Some parents reported that they felt overwhelmed by fear of possible side effects of the MMR vaccination, sometimes after becoming familiar with anti-vaccination campaigners and other parents’ personal experiences. These parents believed that a key skill to be competent in the decision is the ability to assess the reliability of the information received and its quality. For these parents, it becomes difficult to decide which information source to trust. As a consequence, they reported that the decision over the MMR vaccination was emotionally-driven. The mother cited above experienced fear when she was informed, during one of the conferences held by anti-vaccination doctors she regularly attended, about the severe side effects that the vaccination might cause. She described her decision as follows:

*“Since you do not know how these statistics are made and if they are reliable or not, you say: it’s not possible that in the end they reach completely different results. Should I trust one or the other side? And so sometimes… you end up just listening to your gut. […] If you go to one of their conferences, they explain what can happen to the child, they explain everything. And then you start to fear… Because they have interviewed mothers whose children, just after the shot, could not move, or could not speak”.(Mother, 34, EU, Professional University, Opposed)*

Other parents reported that one can perceive himself or herself as competent only when holding accurate information on the MMR vaccination and on the likelihood to catch the disease. Lacking this information, and worried that they could make the “wrong” decision, they did not want to have the final say on it, but preferred to devolve it upon the pediatrician.

*“If I had to guess which percentage of vaccinated children get sick, I don’t know where… I don’t know the percentage. So I would ask the doctor. I’d look on the Internet, but ultimately before making a decision I would ask the doctor anyway”.(Mother, 41, Ticino, University, Favorable)*

Entrusting this decision upon a medical professional without questioning it and refusing to be involved can, however, have dangerous consequences, as some parents also expressed a preference for natural immunity after being recommended by doctors and nurses to avoid the vaccination.

*“To be frank, the pediatrician once told me “There are certain vaccinations that I recommend, others that I don’t. And this is about vaccinating for something that no longer exists, isn’t it? So, honestly, I do not recommend”. And at that time I didn’t know much. I was very busy, too. So I listened to him”.(Father, 28, non-EU, University, Opposed)*

Other parents felt that, since they lacked the training doctors usually have, the MMR vaccination decision could only be driven by one’s family tradition or by social norms related to the original culture. In this case, parents had a propensity for what had been done with them when they were younger, or for what was socially prescribed in their original culture. Participants with an immigration background held a number of health beliefs related to their home healthcare system where vaccination was compulsory or where pro-vaccination social norms were stronger. For these parents, vaccinations in general represented an issue that is never discussed, as immunizations were recommended by a trusted authority. They did not question the importance of the vaccination, as vaccinating was also culturally prescribed in their home country.

*“In Brazil, vaccination is a matter of culture, everyone has his or her own vaccination book, and if you do not fill it, then you are not accepted. It never happens that someone opts out. If there is a vaccine, we just do it. We never discuss about it. We did not study medicine, we just have to trust doctors. […] For me vaccination comes at the first place, possibly because of my culture, this is how I grew up. It is very important to us, to all Brazilians.(Father, 35, Non-EU, Obligatory School)*

Perceived competence in the MMR vaccination decision differed among our participants. Moreover, the idea of competence was also seen by some as related to the ability to make an autonomous decision. Some parents mainly defined it as the set of skills necessary to understand the information provided by official sources (statistics). For others, it is the ability to distinguish reliable from non-reliable information, particularly when contradictory information is presented. Some stated that feeling competent was about being well-informed on the risks and benefits of the vaccination and the risks of the disease(s). For some parents, perceived competence is related to the lack of medical training, and in this case issues of vaccination tradition and social norms can play a strong role, since the decision will be ultimately made in accordance with what was prescribed by the original culture.

### A far-reaching decision

The MMR vaccination decision was generally cited as one of the most significant decisions made since the child’s birth. When asked about what importance meant to them, some parents spontaneously reported that by deciding to give the MMR vaccination to their child they would contribute to accomplishing a global goal and get closer to the eradication of the three target diseases.

*“My main aim is to try to eradicate these diseases. The last time I went to the doctor, I saw a poster that read: in South America, measles has been… I mean, it does not exist anymore. In Switzerland is still present instead”.(Mother, 36, Swiss-German, University, Favorable)*

For others, the vaccination decision is central because it concerns the child’s health. The impact of the decision, in this case, may be of two types: on the one hand, administering the vaccine is perceived as injecting something in the child’s body which might cause harm, while on the other hand, failing to do so might result in the child experiencing a dangerous illness.

*“I did not feel like it, because I felt that I was injecting something harmful. Inside myself, I did not feel like it. So I preferred to listen to these (anti-vaccination) groups. […] But, obviously, I don’t know if he gets measles tomorrow and he dies (as a consequence). This is the most important choice, because it is just about his life”.(Mother, 34, EU, Professional University, Opposed)*

Further support for the importance of this choice rests on some parents’ experience that deciding over the MMR vaccination might affect not only the child’s social life, but also the family life-style.

*“I think that taking him to the nursery school is the most important decision, the one that has the greatest influence on his life right now. But the vaccination is important alike, because it also affects our travel plans. We are frequent travelers, we often go to Africa or Asia”.(Mother, 34, EU, Professional University, Favorable)*

Some parents reported that complying with official vaccination recommendations is a matter of common good and respect towards society, and in this sense they suggested that educational institutions and health authorities should adjust their vaccination policies in order to prevent free-riding and putting at risk those children who cannot be vaccinated for medical reasons.

*“To my mind there should be one guideline. There should be a model that regulates the admission of children with certain requisites at school. Because I think that, if your child is vaccinated, it also protects the others. So it seems to me that this has a scope… a bit more social […] I say… respect! You cannot have everything, you cannot decide this and later exploit public structures where there are norms, right? This is inconsistent to my mind”.(Mother, 40, EU, University, Favorable)*

Parents reported that this social aim is indeed missing among anti-vaccination parents, who merely worry about their own children in an individualistic fashion.

*“For them* [vaccination opposed parents] *it is not important that, unless everyone is vaccinated, we get the disease. I mean, the collective scope, they do not even consider that. They look at their child and say - this way is better, to our opinion”.(Mother, 35, EU, University, Undecided)*

Some saw the concept of importance as a synonym of contingency and stress. In this sense, the MMR vaccination decision was seen as a less compelling choice than others, which instead required a long and constant mental reasoning.

*“I don’t know if I would say that it is more or less important than deciding over the nursery school… It is definitely less pressing, in the sense that choosing over the nursery school has demanded a more careful consideration than deciding on the vaccination, because its consequences were just more contingent. Vaccinating takes a moment. Deciding whether to send him to the nursery, for how many days, which days and so on, that entails a number of choices that go beyond the contingency of the day of the vaccination. It’s a matter of daily life, it’s not just confined to a specific moment”.(Father, 31, EU, University, Favorable)*

In sum, the importance of the MMR vaccination decision is seen by parents in terms of its impact on three main levels: (a) the child’s health, since he/she is the direct recipient of the vaccination, (b) the family’s life-style, as diseases might impede normal activities and habits, and (c) a global/social level, since vaccination is seen in relation to the eradication of the disease and to illness-prevention among the child’s peers.

### The demand for shared decision-making

A main finding is that pediatricians were perceived as key elements in the decision-making process, as both a source of information and motivation to engage in the decision. Although the pediatrician was cited as the main source of information by all parents, differences emerged in term of perceived reliability, adherence to and type of recommendation offered. One quarter of the participants, for instance, reported they were not recommended by the pediatrician to vaccinate against measles.

*“The pediatrician has advised me against MMR. He told me he is not really in favor of vaccinations. But I decided I will do it. I have decided to go against the tide!”(Mother, 35, EU, Secondary School, Favorable)*

For most participants, irrespective of their attitude towards the vaccination, previous consultations with the pediatrician around the topic of the MMR vaccination were not perceived as helpful and often left them frustrated, while more involvement from the health authorities’ side was also claimed. A number of parents complained that they did not receive quality and tailored advice according to their own skills, neither were they directed to reliable information sources.

One mother, for instance, stated that she was dismissed by the pediatrician, who simply recommended her to get informed and return to his office once she had made a decision:

*“When I had to decide for the first vaccination, he told me «Look, I am not so in favor of vaccinations. Look for information and make up your mind. » […] I wish I had clearer explanations, especially because what you read is so vast and hard to interpret. I definitely wish I had better information from the pediatrician. […] He did not even direct me to any sources, he only said “Do as you like”. I wish I had a guide instead”.(Mother, 35, EU, Secondary School, Undecided)*

Some reported that, in order to feel more confident when making the MMR vaccination decision, they would like pediatricians to devolve more time to explaining the risks of the vaccination to parents, by giving a proper lecture on this topic.

*“I think that each pediatrician should set a meeting with all parents and give a proper lecture on this vaccination, not only he alone, but with other doctors. He should give a one-hour lecture, where he explains what he usually does, what he gives to babies when they have this or that problem, where he explains the most common adverse events… I want him to do it so that we feel confident about our decision”.(Father, 28, Swiss-Italian, University, Opposed)*

Parents not only suggested that pediatricians should organize regular consultations with them to answer all questions and explain the possible side-effects of the vaccination, but also expressed a desire to attend meetings with both pro- and anti-vaccination doctors, where they could actively participate in the debate.

*“It would be great if the Canton, or the Confederation, could organize conferences with pro and anti-vaccination doctors, where parents can go and ask all kinds of questions… Because when you go to pro-vaccination events you hear something, and when you go to anti-vaccination conferences you hear something else”.(Mother, 34, EU, Professional University, Opposed)*

In terms of discussion, many felt that a lack of debate was a major weakness of their consultation with the pediatrician. One mother reported that the pediatrician did not engage in discussion on the MMR vaccination with her, and that was among the main reasons why she and her husband were considering switching to another pediatrician:

*“We have never discussed the MMR vaccination with the pediatrician, he only told us there is this vaccine. That’s why my husband and I are challenging him right now… […] He will probably give us only some material to read when we see him next time”.(Mother, 32, EU, University, Undecided)*

Some parents expressed the desire to make an autonomous decision, but at the same time being guided by the pediatrician’s advice and his/her engagement in discussion with the couple of parents. They felt that competence in such a decision could only be achieved through the pediatrician’s guidance.

*“I would like to feel a stronger engagement by the doctor, to receive adequate information, to have a discussion with my husband and, currently, a discussion with both in the same place. […] I feel I can decide, but only if guided by someone in the field, by his or her advice”.(Mother, 27, EU, University, Favorable)*

In addition, for some parents, it is not sufficient that pediatricians simply explain the risks and benefits of the MMR vaccination, but it is important that they take a stand on the topic and state their position.

*“I think pediatricians should take a stand… and if they don’t, we should force them to do it. Doctors will obviously say, “It’s your decision, I just explain the risks and benefits”, but for me it’s important that in the end… how to say… they explicitly take a stand”.(Father, 31, EU, University, Favorable)*

Parents complained that they did not receive quality and tailored information by the pediatrician nor were they directly supported in their information-seeking. In addition, what should probably be the core of a shared decision making approach by the pediatrician, i.e. discussion, was reported by most parents as the biggest deficit of the consultation.

## Discussion

The aim of this study was to qualitatively explore parents’ vaccination literacy and psychological empowerment in the MMR vaccination decision-making in the Canton of Ticino, Switzerland. Since the administration of the first dose of the MMR vaccination is recommended in Switzerland when the child turns 1-year-old, we conducted semi-structured interviews with parents of children aged less than 12 months residing in the Canton of Ticino. This helped prevent making erroneous observations that are likely to occur if one asks decision criteria after a decision was made, for cognitive dissonance theory [81] suggests parents might forget the reasons that guided their decision or justify their behavior on the basis of their later experience.

Regarding vaccination literacy, our results showed that several parents spontaneously reported a belief that the non-compulsoriness of the MMR vaccination in Switzerland is the result of the low likelihood to catch measles that the country enjoys. Furthermore, some parents believed that Switzerland has both compulsory and recommended vaccinations, and translated the non-compulsoriness of the MMR vaccination as further evidence that it was not a necessary preventive measure. This belief can be explained by the fact that some European countries still have both mandatory and compulsory vaccinations. Thus, vaccination literacy has to entail, among other skills such as factual knowledge on the risks and benefits of the vaccination, a correct understanding of the scope of current vaccination policies, since these parents questioned the need for vaccinating [82]. Parents’ misinterpretations of the aims of the recommended vaccination schedule might be linked to parents’ lower risk perception of the disease, which has often been reported among the main predictors of vaccination behavior [10, 12–14, 60, 83], to a refusal of the official schedule or the adoption of unconventional and unsafe preventive measures. Paradoxically, while current vaccination policies are meant to empower parents to facilitate an autonomous decision (e.g. by means of the free choice), parental misinterpretation of the freedom to decide over the vaccination sets the basis for a dangerous self-management of the child’s health. It follows that, if current empowerment strategies are not combined with the promotion of vaccination literacy (i.e. the understanding of current policies and the acquisition of accurate information on the benefits of the MMR vaccination and the risks of contracting diseases such as measles, mumps or rubella), parents are likely to underestimate the benefits and opt for alternatives that clash with official recommendations.

Regarding psychological empowerment, themes related to autonomy (or self-determination) and perceived competence emerged. We found that, in line with previous findings [84], parents’ perception of lacking expertise about the MMR vaccination and its target diseases, their inabilities to understand medical information, and their perceived incompetence in assessing the reliability of the information encountered may constitute a barrier to their active participation in the decision-making and, thus, to an autonomous decision. Our findings indicate that a perceived lack of knowledge on the MMR immunization and the target disease(s) led some parents to completely devolve their decision on the pediatrician, giving up their self-determination. For other parents, social influences might play a central role when they do not believe to be competent in making the decision themselves, with some parents opting for a culturally-embedded decision or for what has been previously done within the broader family [85]. However, our findings add that parents with a low perceived competence might nevertheless opt for an autonomous decision. In this case, we found that some parents had a preference for a gut-driven choice and that this, in turn, could be influenced by feelings of fear and anticipated regret derived from attendance of anti-vaccination meetings. Perceived competence and self-determination appear then to be unrelated. In some cases parental decision will lack both the self-determination which characterizes an autonomous choice and the perception of being competent to make an informed decision, two characteristics currently advocated by vaccination policies. In other cases, the perception of being unable to make a decision does not constitute an obstacle to parents’ self-determination, who might follow their own instinct and make an autonomous decision, running the risk of being at the mercy of anti-vaccination supporters or old-fashioned and unsafe beliefs. This might have serious implications for the formation of beliefs on the safety of the MMR vaccination, since most anti-vaccination narratives include, for instance, stories of children who allegedly became autistic after receiving the MMR jab [86, 87].

Regarding importance (or meaningfulness) and impact, two sub-dimensions of psychological empowerment, it appears in the results that the MMR vaccination decision is listed by all parents among the most important decisions made for their child, including parents who have a negative attitude towards the MMR vaccination, who mainly worry about the vaccine’s side effects on the child. This is in line with previous studies [53, 88]. However, while importance comes as an obvious component of the decision, independently of the attitude towards the vaccination, this theme is enriched by the finding that parents’ concerns address three main levels which the MMR vaccination decision can have an impact on, namely the child’s life, the family life-style, and the community/society. Commitment to preserve one’s child and other children from the disease was found to be a predictor of parental vaccination decision [15], as well as parental concerns about a vaccination decision affecting family life-style [89], and these issues are mainly cited by pro-vaccination parents. For some parents, importance was conceived in terms of contingency and stress, in the sense that the MMR vaccination decision was deemed less important than other choices since it did not require long and constant organizational efforts.

In their quest for vaccination literacy and psychological empowerment, most parents seem to find a potential and desired ally in the pediatrician. Parents’ expressed a need for shared vaccination decision-making with the child’s healthcare provider, and this is in line with previous studies that reported discussion with a doctor was associated with receipt of the vaccination [90, 91]. Ideally, shared decision making (SDM) in the context of childhood vaccination decision would be characterized by the pediatrician explaining the risks and benefits of the vaccination according to the individuals’ competences, listening to parents’ preferences, and discussing the decision with both parents so that the decision is informed and made in accordance with parental values and needs [45]. Parents mainly advocated more discussion with the pediatrician prior to making the final decision as a way to be more involved and, thus, build their path towards a self-determined choice. Also, they asked to receive quality and tailored information on the risks and benefits of the vaccination according to their skills, which will lead to a perceived competent choice. Finally, they want the pediatrician to provide factual and procedural information, and tools to find, access, and understand this information, which is in line with what vaccination literacy would entail. Many parents felt that they could make an empowered decision, but that this did not mean being entirely independent and without the pediatrician’s advice. Rather, they felt the need for an expert guide to better understand the risks and benefits of the vaccination, in order to make a choice that could ultimately be driven by themselves. This partly contradicts Opel’s and colleagues’ [42] finding that pediatrician’s communicative style (presumptive vs. participatory) was related to vaccination receipt, in the sense that a presumptive approach was found to be correlated with higher compliance. This difference can be explained by cultural differences, as Opel’s study was conducted in the US. A similar study should be conducted in Switzerland to assess whether parental needs for shared decision-making are in accordance with preference for a presumptive or participatory style by the pediatrician.

Limitations of the currents study include that parents who accepted to participate in the study were most receptive to the topic of childhood vaccinations and more prone to discuss their experience and position. Due to the qualitative nature of the present study and its limited sample size, it should also be stressed that our findings cannot be generalized to the whole population. Moreover, social desirability bias should be taken into account, since participants might be more prone to present themselves as compliant with official recommendation, especially when they mentioned that adherence to the vaccination schedule for their older children was meant to secure immunity within their community. Furthermore, since the Italian-speaking part of Switzerland has a higher MMR vaccination coverage compared to the rest of the country [6], exploring the concepts of vaccination literacy and empowerment in a low coverage area might have yielded different insights. However, our diverse recruiting system helped us minimize these limitations ensuring a diversified sample in terms of country of origin and life-styles. Parents with an immigration background represented a large percentage of our sample. However, this is in line with current statistics on the migrant population in Switzerland. Moreover, as qualitative research is context-bound, parental reports are to be interpreted according to the Swiss context and healthcare system that immigrants necessarily navigate and integrate with their past beliefs. Qualitative research may represent an effective tool to understand health practices at the local level.

Our study stresses the importance of vaccination literacy and empowerment in the MMR vaccination decision making and, most importantly, of pediatricians as both literacy and empowerment providers during such a decision. First, if parents are given permission to participate in the decision, then the matter to be decided (vaccinating or not) appears to them to be unimportant - this seems to be an important and so far undiscovered and unwanted side effect of psychological empowerment. Second, the participants seem to be quite aware of their low competence in deciding about vaccination. If parents do not feel that they have the knowledge and the skills (in other words, the literacy) required to make a decision on their own, they will delegate other stake-holders to determine their choice, giving up their self-determination and, worse, running the risk of devolving the decision to anti-vaccination actors. Third, parents also seem to be quite aware of the tension between low literacy and high empowerment, mainly because they wish for more participation of pediatricians. This points to the interesting part that people share some understanding of the central premise of the health empowerment model: namely that high empowerment not accompanied by a high literacy is a dangerous thing.

## Conclusions

Our results yield a number of implications at multiple levels. A first level is more concerned with the medical encounter between parents and pediatrician, where the vaccination issue is addressed and discussed. Building on the needs that parents articulated in this study, there are a number of practical implications for pediatricians. Pediatricians should involve both parents in the decision-making, providing the proper information, motivating them to be active actors in this choice, and highlighting the importance of parental role in managing their children’s health as a way to reach empowerment. However, attention should be paid to their communicative style during vaccination recommendation [42]. They should also stress that the importance of their decision lies in the non-compulsoriness of the vaccination, a policy that can be justified neither by a low risk of measles nor by a high risk of experiencing MMR-related side effects, but which is aimed at increasing their sense of responsibility and empowerment. Strategies to empower parents might include discussing the impact of the decision at the child, family and collective level, highlighting possible negative consequences of non-immunization. Concerning vaccination literacy in the specific, pediatricians are urged to provide clear, concise and tailored information regarding the risks and benefits of the MMR vaccination in a format that parents can understand and process. They should be able to counter-argue inaccurate arguments regarding the risks posed by the vaccination and those posed by the diseases that the vaccination aims at preventing, highlighting the disadvantages of missed or late pediatric immunizations. Lastly, they should be prompt in directing parents to reliable, accessible and clear information sources, before they fall victim of inaccurate information disseminated by anti-vaccination advocates, which is usually preferred for its narrative style. However, it should be stressed that following these recommendations may represent a challenging task for pediatricians, as being more actively engaged would inevitably require more work time – a limited resource.

Further implications of our results rest at a policy and institutional level. Policy-makers are urged to explicitly disclose the rationale behind the non-compulsoriness of pediatric vaccinations. This could be done by stressing the democratic and ethical character of the country’s health related policies, or the thrust to positively engage parents and make them responsible for their children’s health.

At a research level, further exploratory or conclusive research is needed to better understand the extent to which being literate and empowered contribute to the MMR vaccination decision-making. In particular, psychological empowerment deserves a deeper investigation in a population where vaccination rates are low, and measurement issues should be addressed to provide tools to quantitatively assess parental empowerment in making a MMR vaccination decision for their children.

Since parents are expected to make an informed and autonomous decision regarding their children’s immunization, successful communication with respect to childhood vaccinations, and the MMR vaccination in particular, should take into account both issues of vaccination literacy and psychological empowerment. Healthcare providers and health authorities should promote parental empowerment as a process through which parents gain control and responsibility over the health decisions they make for their children, especially with regards to their immunization schedule. This could be done by highlighting the significance and the potential impact of the decision, and the importance of being literate on the topic to feel competent and autonomous. Efforts should be made, on the one hand, to give parents the proper information about the vaccination and the target disease(s), but also the skills to find more information, to assess its reliability and, ultimately, to understand it. This can in turn increase parents’ perception of being competent and thus make an empowered decision.

## Additional file

Additional file 1:
**Interview schedule.** List of questions designed to be asked during the semi-structured interviews. The questions do not necessarily appear in the order in which they were to be asked. (PDF 122 kb)
