# Supplementary material for: Addressing issues of vaccination literacy and psychological empowerment in the measles-mumps-rubella (MMR) vaccination decision-making: a qualitative study
Source: BMC Public Health. 2015 Sep 2;15:836. doi: 10.1186/s12889-015-2200-9 (PMC4556054; doi:10.1186/s12889-015-2200-9)
Supplement: Additional file 1: — Interview schedule. List of questions designed to be asked during the semi-structured interviews. The questions do not necessarily appear in the order in which they were to be asked. (PDF 122 kb) [file 12889_2015_2200_MOESM1_ESM.pdf]

Appendix 1. Interview schedule

Interview topics and sub-topics	Key interview questions
1. Confidence in one's MMR vaccination decision	→ How confident are you in your decision about the MMR vaccination for your child?
2. Vaccination literacy	
General beliefs	→ What do you think of the MMR vaccination?
Procedural knowledge	→ When is MMR due for your child?
Subjective knowledge	→ Do you feel sufficiently informed about the MMR vaccination?
Perceived outcomes of MMR	→ What comes to your mind when you think of the positive outcomes of the MMR vaccination?
	→ What comes to your mind when you think of the negative outcomes of the MMR vaccination?
Information seeking behaviors	→ Which sources did you use?
	→ Which sources have contributed most to your decision-making?
3. Empowerment	
Meaningfulness	→ What are the major decisions that you have made for your child so far?
	→ How important is your choice about the MMR vaccination compared to other decisions made for your child so far?
Self-efficacy	→ What makes one able to make a sound decision about MMR?
	→ What skills would one need to have in order to feel able? What does one need to know?
Self-determination	→ In your opinion, what does it mean to be autonomous in making a decision regarding MMR?
Impact	→ Under which circumstances would you feel that your decision regarding MMR did make a difference?
4. Social influences	→ Have you talked about the MMR vaccination with someone?
	→ Where and with whom?
	→ What have you been told?
5. Reactions to MMR-related information	→ Think of the last time you came across information on the MMR vaccination. Was there any information that made you particularly scared?
	→ Was there any information that made you particularly secure and relieved?
6. CAM usage	→ Do you use complementary or alternative medicines (CAM)?
	→ What comes to your mind when you think of CAM and measles?
7. Perceived risk of MMR and measles	→ What comes to your mind when you think of the probability that your child will contract measles?
	→ What comes to your mind when you think of its severity?
	→ What comes to your mind when you think of the probability that your child will have MMR side effects?
	→ What comes to your mind when you think of their severity?
8. Barriers to the decision	→ Was there anything that frustrated you during your decision-making?
